# Transitioning between the EQ-5D youth and adult descriptive systems in a group of adolescents

**DOI:** 10.1186/s41687-024-00770-4

**Published:** 2024-08-12

**Authors:** Janine Verstraete, Paul Kind, Mathieu F. Janssen, Zhihao Yang, Elly Stolk, Abraham Gebregziabiher

**Affiliations:** 1 Department of Paediatric and Child Health, University of Cape Town, Cape Town, South Africa; 2 University College London, London, UK; 3 EuroQol Research Foundation, Rotterdam, The Netherlands; 4 Health Service Management Department, Guizhou Medical University, Guiyang, China; 5 Mekelle University, Mekelle, Ethiopia

**Keywords:** EQ-5D-3L, EQ-5D-5L, EQ-5D-Y, Transition, Adults, Preference-weighted scores

## Abstract

**Purpose:**

To investigate whether the same health state results in the same distribution of responses on the EQ-5D youth and adult descriptive systems.

**Methods:**

Adolescents aged 13–18 years with a range of health conditions and from the general school going population were recruited in South Africa (ZA) and Ethiopia (ET). In ZA participants completed the English EQ-5D-3L, EQ-5D-Y-3L and EQ-5D-5L in parallel. Whereas in ET participants completed the Amharic EQ-5D-5L and EQ-5D-Y-5L in parallel. Analysis aimed to describe the transition between youth and adult instruments and not differences between countries.

**Results:**

Data from 592 adolescents completing the EQ-5D-3L, EQ-5D-Y-3L and EQ-5D-5L (ZA) and 693 completing the EQ-5D-5L and EQ-5D-Y-5L (ET) were analysed. Adolescents reported more problems on the youth versions compared to the adult version for the dimension of mental health. 13% and 4% of adolescents who reported no problems on the EQ-5D-3L and EQ-5D-5L reported some problems on the EQ-5D-Y-3L respectively. This was less notable with transition between the five level versions with 4% of adolescents reporting more problems on the EQ-5D-Y-5L than the EQ-5D-5L. Very few adolescents reported severe problems (level 3 on the EQ-5D-3L or EQ-5D-Y-3L and level 4 and level 5 on the EQ-5D-5L or EQ-5D-5L) thus there was little variation between responses between the versions. In ZA, discriminatory power, measured on the Shannon’s Index, was higher for Y-3L compared to 3L for pain/discomfort (ΔH′=0.11) and anxiety/depression (ΔH′=0.04) and across all dimensions for Y-3L compared to 5L. Similarly, in ET discriminatory power was higher for Y-5L than 5L (ΔH′ range 0.05–0.09). Gwet’s AC showed good to very good agreement across all paired (ZA) 3L and (ET) 5L dimensions. The summary score of all EQ-5D versions were able to differentiate between known disease groups.

**Conclusion:**

Despite the overall high levels of agreement between EQ-5D instruments for youth and for adults, they do not provide identical results in terms of health state, from the same respondent. The differences were most notable for anxiety/depression. These differences in the way individuals respond to the various descriptive systems need to be taken into consideration for descriptive analysis, when transitioning between instruments, and when comparing preference-weighted scores.

**Supplementary Information:**

The online version contains supplementary material available at 10.1186/s41687-024-00770-4.

## Background

The EQ-5D family of instruments are generic patient reported outcome measures used to inform clinical and economic decision making [1]. The adult and youth versions of the instruments are used globally across a range of disease groups and settings. The EQ-5D-Y-3L (Y-3L) was developed from the original adult version, EQ-5D-3L (3L), using child friendly wording [2]. This Youth version was later expanded to include five levels of severity, EQ-5D-Y-5L (Y-5L) with similarities to the adult EQ-5D-5L (5L) [3]. EuroQol’s current guidelines state that adolescents aged 15–18 years can be asked to administer either the youth version or the adult version depending on the study characteristics [1]. The choice of adult or youth instrument may however influence the responses and subsequently the preference-based values used for health decision making. There may further be longitudinal studies in which transition between youth and adult instruments is unavoidable. Furthermore, one may want to aggregate or compare adult and youth results within a health condition or between health conditions.

Limited evidence exists about how responses differ between the EuroQol youth and adult versions. Results from the multi-national study on the development [2] and validation [4, 5] of the Y-3L included children aged between 8 and 18 years from school samples in Germany, South Africa (ZA), Spain and Sweden. Germany, Spain and ZA further compared the Y-3L to the adult version and found a reduced ceiling effect and less missing responses in the youth version. The ZA results found that the Y-3L showed more reported problems than the 3L, although it must be noted that these versions were however completed by different participants [5]. A study in US adults found that the ceiling (11111) was lower for the Y-3L than the corresponding adult version [6]. The advent of the 5L and Y-5L now allows further transitions.

To understand how outcomes in the transition period using EQ-5D (including both 3L and 5L) and EQ-5D-Y (including both Y-3L and Y-5L) compare, we need to understand how reporting on these descriptive systems changes. We anticipated that most transitions would occur between the versions with the same number of levels (Y-3L and 3L or Y-5L and 5-L). However, as the Y-5L is newly developed [3] there may be studies where transition from the Y-3L to the 5L is necessary. Thus, this study aims to determine how the differences in wording on descriptive systems of the adult and youth version (Y-3L vs 3L, Y-3L vs 5L and Y-5L vs 5L) impact the transition between instruments.

## Methods

### Study design and participants

An observational, cross-sectional study was conducted in adolescents aged 13–18 years in South Africa (ZA) and Ethiopia (ET). To avoid confounding transitional effects and linguistic differences, we separated transitions of interest per country. In ZA the transitions from Y-3L to 3L and Y-3L to 5L were investigated whereas in ET the transition from Y-5L to 5L was explored. Participants were recruited across a range of health conditions in both countries to ensure a range of illness severity and thus health states selected on the EQ-5D versions. Comparison between country data was not of interest.

#### South African setting

Adolescents with HIV/AIDS, cardiac disease, diabetes mellitus and respiratory illness were recruited from adolescent clinics at either a specialist adult or paediatric hospital in Cape Town, South Africa. Adolescents with functional disability were recruited from schools for learners with special educational needs (LSEN), who follow a mainstream curriculum, in Cape Town, South Africa. Participants were further recruited from the general school going population from schools in the same geographical area as the hospitals and LSEN schools.

#### Ethiopian setting

Adolescents with cardiac disease, respiratory illness, HIV/AIDS and diabetes mellitus were recruited from their corresponding clinics and adolescents with functional disability were recruited from the physiotherapy unit and medical clinic of Tikur Anbessa Specialized Hospital in Addis Ababa, Ethiopia. Tikur Anbessa Specialized Hospital, the largest teaching hospital under the administration of Addis Ababa University in Ethiopia. The general school going group of adolescents were recruited from different government (elementary and high school) schools in Addis Ababa city, Ethiopia.

### Instruments

#### EQ-5D instruments

The official Y-3L, 3L and 5L versions and experimental Y-5L versions were used in this study (10). All versions have been validated in ZA [7–10] and ET [11]. Although the English, Xhosa and Afrikaans versions were made available in ZA, all participants chose to complete the English version. The Amharic versions were used for ET. Each of the instruments include five dimensions with slightly different wording; mobility, self-care or looking after myself, usual activities, pain/discomfort and anxiety/depression or feeling worried, sad or unhappy (Supplementary Table 1). In addition to the changes reflected in Supplementary Table 1 the Amharic Y-5L version for ET with an adaptation for pain/discomfort to “having pain or physical discomfort (for example, itching, dizziness, or feeling sick)” with response levels referring to pain or physical discomfort too.

The health dimensions are scored on either three levels or five levels of severity (Supplementary Table 1) [11]. For example the Y-3L is scored as 1 = no problems, 2 = some problems and 3 = a lot of problems. The three or five levels of the descriptive system are expressed with a five-digit code. For example, the Y-3L health state 11223 describes someone with no problems with mobility, no problems with looking after myself, some problems with usual activities, some pain/discomfort and very worried, sad. The best health state described by the instrument is coded as 11111, describing ‘no problems’ in all dimensions [18].

Although the 3L, 5L and Y-3L have preference-based scores the Y-5L does not [19–23]. As such a level sum score (LSS) was used to describe the responses on the descriptive system where the level labels are treated as numeric data with the best possible score (1 + 1 + 1 + 1 + 1) = 5 and the most severe score for the three level versions is (3 + 3 + 3 + 3 + 3) = 15 and five level versions is (5 + 5 + 5 + 5 + 5) = 25. This is a crude measure with limitations but gives some indication of the performance of the dimensions between versions.

### Preference of version

Questions to explore preference of the adult and youth versions in ZA (3L vs Y-3L only) and ET (5L vs Y-5L) were included. The questions included:

- Which response scale/questionnaire did you find easiest to use? Why?
- Which response scale/questionnaire do you think best expresses your experience? Why?
- Considering the item heading on the two questionnaires which did you prefer? Why?
- Considering the response options on the two questionnaires which did you prefer? Why?

### Procedure

Ethics approval was obtained from the University of Cape Town, Faculty of Health Sciences, Human Research Ethics Committee (HREC 839_2020) and ethics review committee of Addis Ababa University college of health science, school of pharmacy research and community service office (ERB/SOP) (ERB/SOP/241/13/21). The study was carried out in accordance with the declaration of Helsinki involving human participants [12].

All data was collected using a pen-and-paper survey and different versions of EQ-5D were presented in random order. In ZA adolescents completed the English Y-3L, 3L and 5L whereas in ET they completed the Amharic Y-5L and 5L Administration of the EQ-5D versions were separated by an age-appropriate demanding cognitive task/problem to reduce bias. After completing the survey, the adolescents completed questions to help understand which version was more suitable for the 13-18-year-old group. Demographic and medical information was also captured.

Due to the constraints of the Covid pandemic in ZA children/adolescents from the LSEN and general school going population were recruited through information leaflets that were sent home to them and their parents. For those who were willing and provided consent and assent the instruments were self-completed by the adolescent. Depending on the level of Covid restrictions ZA adolescents with a health condition were recruited in person or through recruitment flyers in the respective clinics and self-completed. There was a researcher available for questions of clarification in the clinical setting in ZA and in all settings in ET.

### Data management and analysis

Statistical analysis was conducted using Stata Version 14.0 SE. The EQ-5D responses and descriptive data were summarised in terms of frequency of responses. The ceiling of all versions was examined in those with a health condition and defined as the proportion of adolescents scoring no problems in each dimension or no problems in a dimension across all five dimensions (11111). The ceiling was compared between paired versions with the *X*^*2*^ test and the absolute reduction in proportion scoring was calculated for participants with a health condition.

Paired dimensions responses (Y-3L vs 3L, Y-3L vs 5L and Y-5L vs Y-3L) were assessed for redistribution of responses. The change in levels with transition between instruments was described as the proportion of adolescents who reported discrepant health state.

Following Janssen et al the discriminatory power of all EQ versions were evaluated in terms of absolute and relative informativity evaluated by the Shannon Index (H′) and Evenness Index (J′) [13, 14]. A higher H′ index reflects that the descriptive system has captured more information, the maximum H′ index is 1.58 and 2.32 on the Y-3L and Y-5L respectively. The Evenness index (J′) reflects the spread of the responses across levels regardless of the number of levels included in the descriptive system.

Agreement of dimensions scores on the paired responses (Y-3L vs 3L, Y-3L vs 5L and Y-5L vs Y-3L) were calculated with Gwet’s AC. The Gwet’s AC was used in this sample due to the low variability in health states and provides a more stable statistic than kappa [15]. A Gwet’s AC of < 0.2 was interpreted as poor agreement; 0.21–0.4 as fair; 0.41–0.6 as moderate; 0.61–0.8 as good and > 0.8 as very good.

Known group validity was determined by comparing the mean (SD) LSS scores for each instrument between known health condition groups by ANOVA F-statistic. Comparison between instrument (Y-3L vs 3L, Y-3L vs 5L and Y-5L vs Y-3L) mean (SD) LSS scores were analysed with the Wilcoxon signed-rank test.

Frequency of responses from preference between versions (Y-3L vs 3L and Y-5L vs 5L) were compared with *X*^*2*^ test. Open ended responses for reasoning for responses were coded for thematic analysis. Level of statistical significance was set at *p* < 0.05 for all analyses.

## Results

### Characteristics of participants

**Fig. 1:**
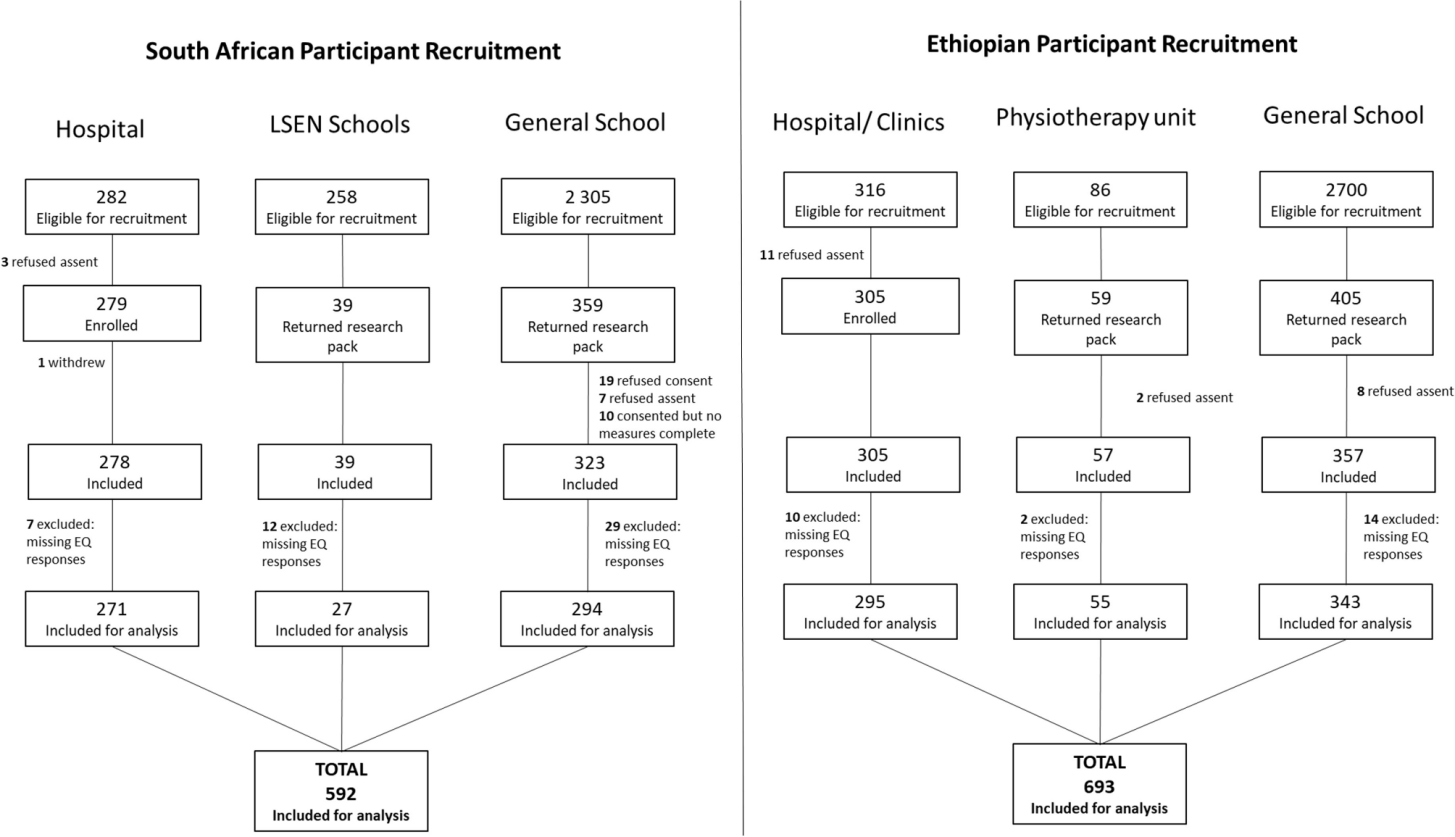
Recruitment into the study

The recruitment and enrolment of participants in ZA and ET are detailed in Fig. 1. The reason for refusal of consent/assent was not collected. One participant withdrew in ZA due to time constraints. Participant data was excluded if there were any missing EQ dimension or VAS responses. A total of 592 ZA participants and 693 ET participants were included for final analysis.

The sample characteristics are shown in Table 1. There were more female (54.9%) respondent in ZA but similar distribution of sex in ET (female 50.8%). There were more adolescent respondents aged 16–17 years in both arms.

### Comparison of general instrument performance

There were slightly more unique health states reported on 3L (*n* = 64) compared to Y-3L (*n* = 61) (*X*^*2*^ = 0.097, *p* = 0.755). Conversely, the Y-5L (*n* = 133) had significantly more unique health states than 5L (*n* = 120) (*X*^*2*^ = 0.696, *p* = 0.040). There was very low reporting of the most severe problems across all instruments and dimensions. The dimensions of anxiety/depression or worried/sad/unhappy had the highest report of problems across all instruments.

Considering those with a health condition the ceiling was significantly higher for anxiety/depression and feeling worried/sad/unhappy for the two adult versions 3L (70.8%) and 5L (69.8%) when compared to the Y-3L (60.4%) respectively (*X*^*2*^ = 7.15, *p* = 0.007), (*X*^*2*^ = 5.79, *p* = 0.016) (Table 2). This variation in reporting of no problems was not however seen when the adult (5L) and youth (Y-5L) five level versions were compared (*X*^*2*^ = 0.01, *p* = 0.007).

The ability of the youth and adult instruments to detecting problems, in adolescents with a health condition, was compared overall by the trend in the total ceiling (11111).The total ceiling (11111) was higher on the 5L (45.3%) than the Y-3L (37.2%) (*X*^*2*^ = 3.99, *p* = 0.046). There were no significant differences between the 5L and Y-5L for total ceiling (11111) (Table 2).

### Redistribution of responses

When considering the transition between the youth and adult instruments the most notable change in distribution of responses was for the mental health dimensions followed by pain/discomfort. The youth versions had more reporting of problems in these two dimensions than the adult versions.

**Fig. 2:**
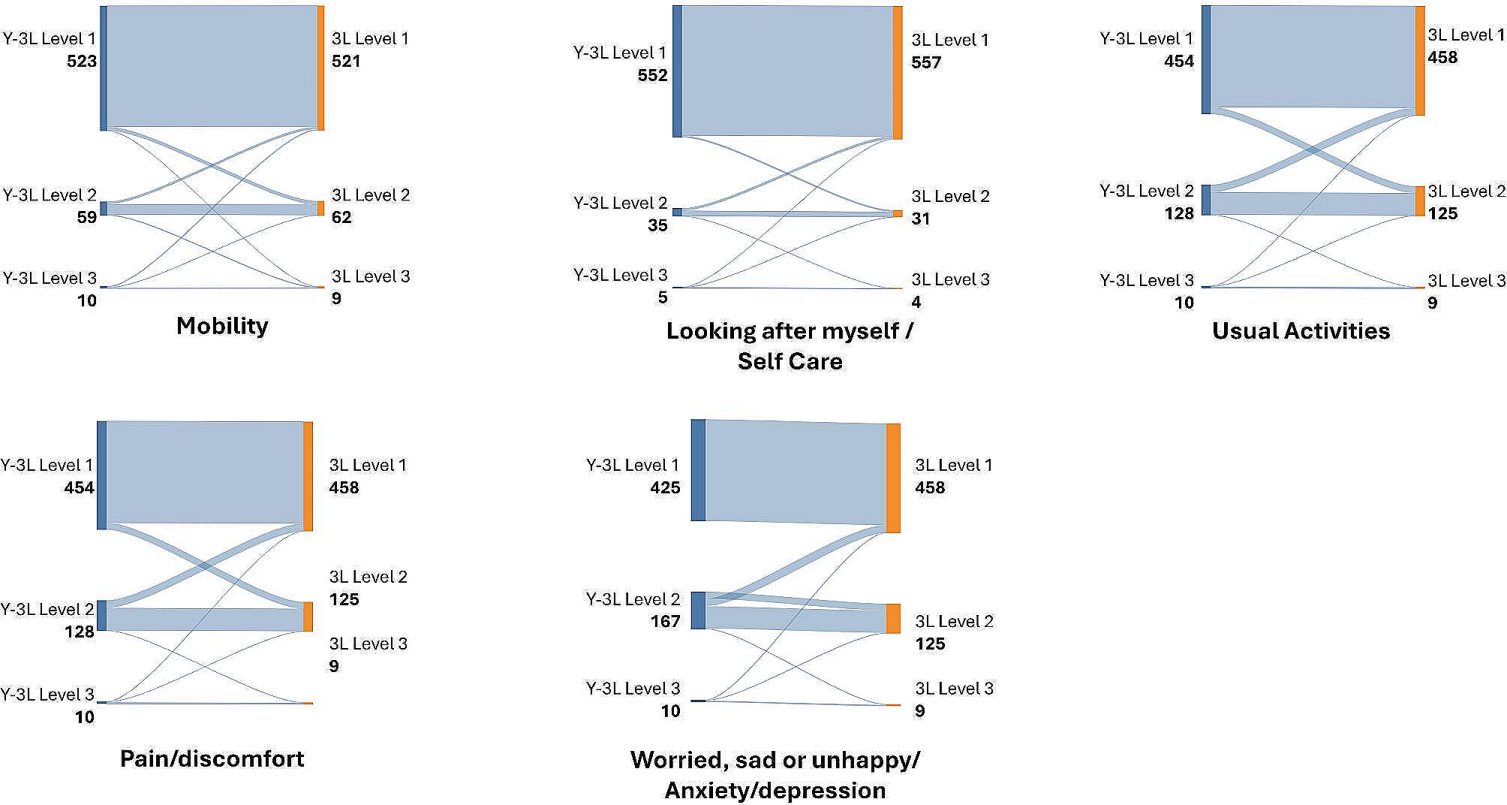
Sankey diagram for EQ-5D-Y-3L and EQ-5D-3L level proportions

When one transitions from the Y-3L to 3L 13% (*n* = 75) of participants reported some problems with mental health on the Y-3L and no problems on the 3L. A smaller number (*n* = 26, 4%) reported some problems with mental health on the 3L and no problems on the Y-3L. Similarly, 8% of respondents (*n* = 45) reported no problems with pain/discomfort on the 3L and some problems on the Y-3L (Fig. 2).

As expected there was generally more variation in responses moving between the Y-3L and 5L. Whereas, the variation in responses between the Y-5L and 5L were less notable.

**Fig. 3:**
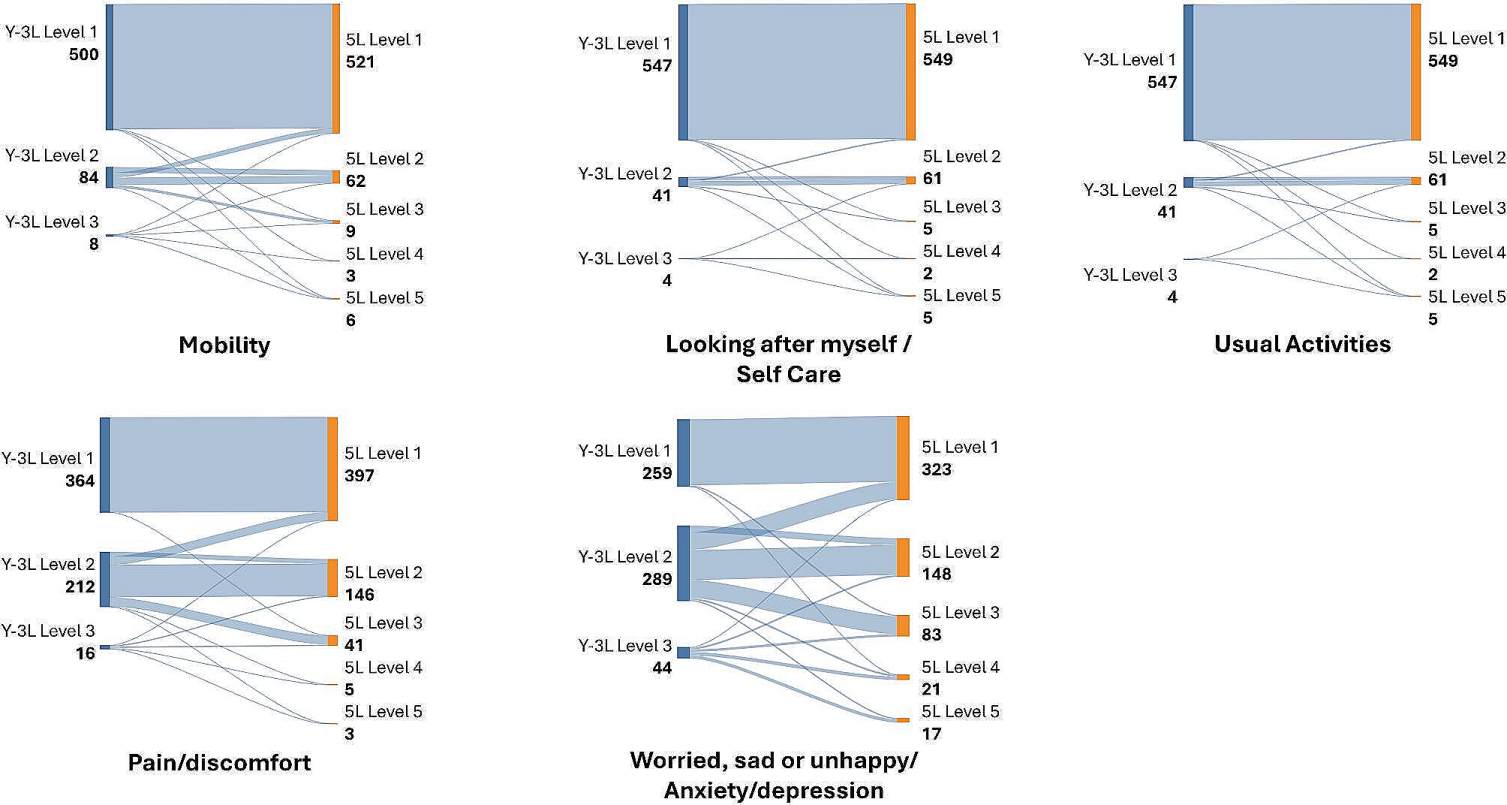
Sankey diagram for EQ-5D-Y-3L and EQ-5D-5L level proportions

**Fig. 4:**
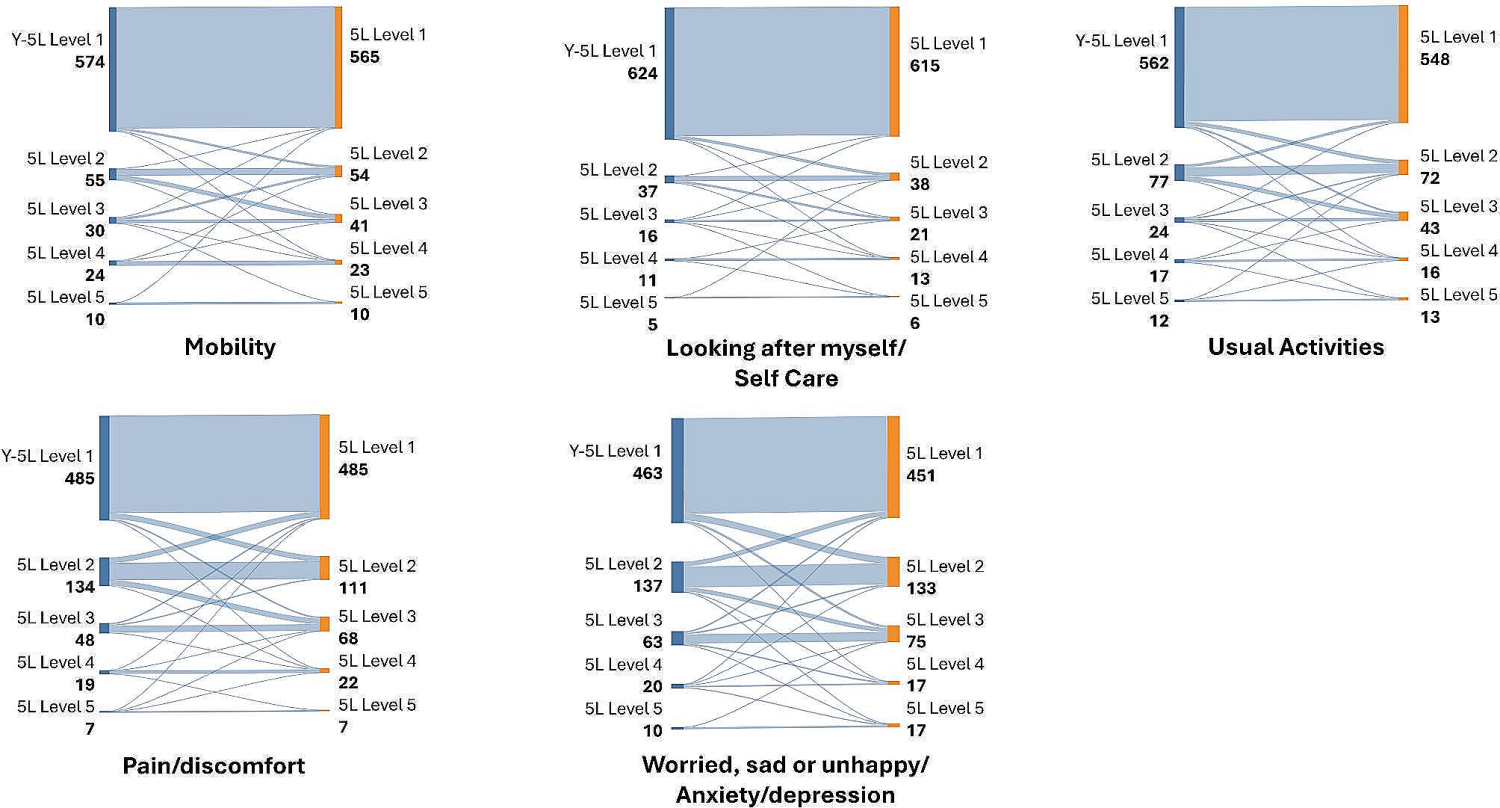
Sankey diagram for EQ-5D-Y-5L and EQ-5D-5L level proportions

Of note in the mental health dimension 11% (*n* = 67) of participants reported no mental health problems on the 5L but some problems on the Y-3L (Fig. 3). In contrast, there were slightly more participants who reported no mental health problems on the 5L and a little bit of problems on the Y-5L (*n* = 30, 4%) (Fig. 4).

### Discriminatory power

The discriminatory power was higher for Y-3L compared to 3L for pain/discomfort (Δ H′=0.11) and anxiety/depression (Δ H′=0.04) and across all dimensions for Y-3L compared to 5L with the largest differences for usual activities (ΔH′=0.32) and anxiety/depression (Δ H′=0.38) (Table 3). Similarly, the discriminatory power was higher across all dimensions for Y-5L compared to 5L (Δ H′ range 0.05–0.09). The distribution of the responses across the instruments was retained with small differences in the evenness index (J′).

### Agreement between instruments

Gwet’s AC showed very good agreement across dimensions of all paired versions (ZA Y-3L vs 3L) and (ET Y-5L vs 5L) dimensions except for anxiety/depression and feeling worried/sad/unhappy on the 3L versions which showed good agreement [0.68 (0.63, 0.73)] (Supplementary Table 2).

### Known group validity

Table 4 shows that comparison of sex and age were insignificant for the LSS of all versions except for age on the EQ-5D-5L in ET (F = 0.421, *p* = 0.015) with a higher mean LSS, indicating worse HRQoL, in the 13–15 year olds when compared to the 16–17 year olds. The LSS of all versions were able to differentiate between known disease groups (school, cardiac, diabetes, functional disability, HIV and respiratory disease). Post hoc analysis (Table 5) showed that the adult 5L LSS was able to discriminate between children living with diabetes and the general school group but the Y-3L was not.

### Preference for the adult or youth instrument

In ZA adolescents expressed a large indifference to the version (Y-3L vs 3L) for completion considering its ease of use, applicability at describing their health state and response options (Table 6). Where a choice was made the Y-3L was preferred. When the dimensions descriptors or headings were considered, it was clear that the adolescents preferred those of the youth version. In ET adolescents had a clear preference for the Youth version (Y-5L) compared to the adult 5L.

Generally adolescents considered both the adult and youth EQ-5D instruments to be easy, understandable, straightforward and/or clear which largely contributed to their preference. Notably the youth versions were considered more personal, relatable or relevant to the adolescent’s health condition contributing to the choice for easiest instrument to use and best description of health experience in both ZA and ET.

The reason behind the preference for dimension descriptors seemed to support the fact that the wording on the youth descriptive system was more detailed or specific and thus easier to comprehend.

## Discussion

Both the ZA and ET arms reported generally mild health states despite inclusion of adolescents with a health condition in each arm. As there is little evidence comparing the adult and youth descriptive systems these results highlight how changes in dimension descriptors and severity labels change the reporting of health states.

The Y-5L had significantly more unique health states than the 5L whereas the difference was not significant between the Y-3L and 3L. This could be attributed to the Y-5L for ET having two dimensions with different wording than the adult comparator e.g. anxiety/depression vs feeling worried/sad/unhappy and pain/discomfort vs having physical pain/discomfort (for example, itching, dizziness or feeling sick). Furthermore, the five levels allow for more variation, corresponding to more unique health states. The difference in unique health states may be of less consequence when transitioning between the youth and adult descriptive systems as there will always be variations in responses due to poor understanding or lack of attention. Considering the change in reporting of health states when moving between the Y-5L and the 5L there was similar movement between level 1 and level 2 for the dimension of pain/discomfort. This change in response options is higher than for the dimensions of mobility, self-care and usual activities and could be attributed to the reference to *physical* pain with examples in the Amharic for ET youth version but not the adult version. The inclusion of examples of pain/discomfort may have further contributed to the higher discriminatory power for the Y-5L compared to the 5L. This discrepancy was not noted with transitions between the Y-3L to 3L and the Y-3L to 5L, where the dimension headings were identical in wording. It is recommended that future work explores this in other language and cultural settings. There may further be a need to harmonise dimensions headings across youth and adult versions.

The change in reporting of mental health was notable in all transitions between the youth and adult descriptive systems with the youth descriptive system reporting more problems. The difference in reporting problems was 13% higher on the Y-3L than the 3-L, 11% higher on the Y-3L than the 5L. The level descriptors between the youth and adult could impact this response shift with the English Youth descriptor on the Y-3L referring to some problems and the adult and 3L and 5L to moderate and slight problems respectively. In contrast there was 4% higher report of problems with mental health on the Y-5L than the 5L. This may be attributed to the label descriptors used in Amharic (ET) where the identical descriptors were used in both versions for level 1, level 3 and level 4. Level 2 were arguably more similar on the Amharic version, when compared to the English version, where the youth version referenced very little problems and the adult versions little problems. The contribution of the descriptor heading to the reporting of problems in mental health is less certain. The English (ZA) and Amharic (ET) versions both refer to feeling worried, sad or unhappy on the youth version and anxiety/depression in the adult version. Future work needs to explore the transition between the English versions of the Y-5L and 5L, the source version for translations.

For both ZA transitions (Y-3L vs 3L and Y-3L vs 5L) the highest discrepancies were attributed to the most severe anchor (3/5) which were semantically different with the youth version referencing “a lot/very/extremely’ compared to ‘cannot/confined to bed/extreme”. There were ten adolescents who reported that they were confined to bed on the 3L, to note eight of these responses redistributed to less severe levels on the Y-3L. It is recommended that future work should target participants known to have severe restrictions in mobility to further explore this difference. In general the more physical dimensions of mobility, self-care/looking after myself and usual activities had lower variation in report, smaller difference in discriminatory power, and higher agreement in responses between versions. This was similarly reported in comparison of 3 level and 5 level adult (3L vs 5L) or youth instruments (Y-3L vs Y-5L) [10, 13] and between the Y-3L and 3L in a ZA school sample [5] and could be attributed to the stability of these physical dimensions. However, this could also be due to the relatively higher ceiling in this sample and/or considering that the dimension descriptors were most similar for these dimensions with differences in the severity labels. One may argue that the severity labels are less problematic when completing the descriptive system as the responses may be chosen due to their relative position in response options and not necessarily due to the semantic description [16]. This effect may need to be explored further in preference-weighting tasks where levels are presented outside of this context.

The ceiling was only considered in those with a health condition at a dimension level (no problems) and a composite level (11111) as one would expect a generally healthy population, such as a general school sample, to have a high ceiling and they were thus excluded. Similar to results from the US there was no significant difference in ceiling (11111) between the Y-3L and 3L [6]. There was a significant decrease in ceiling (or higher reporting of problems) on the Y-3L version when compared to the adult 3L and 5L versions for anxiety/ depression and feeling worried/sad/unhappy. This may indicate that the difference in dimension descriptors contributes to greater reporting of problems than a higher number of response options. At a composite level the total ceiling (11111) was reduced with the increase in response option on the adult version (Y-3L vs 5L). There were no significant differences in ceiling noted when transitioning between theY-5L and 5L with most changes occurring between level 2,3 and 4.

The LSS gives an indication of the composite performance of the dimensions in the absence of preference-weighted scores in these settings. Both the adult and youth versions performed well and were able to detect significant differences between known groups. The Y-3L was better able to discriminate between groups which traditionally report milder health states on the EQ descriptor system including a general school sample [4, 5, 9, 17], HIV [18] and respiratory groups [13] when compared to the adult 3L and 5L. When considering the increase in levels on the adult 5L these showed better ability to detect problems, compared to the Y-3L for the school and HIV groups only. Similarly the Y-5L was better able to identify problems in a general school sample, cardiac and functional disability groups. It is unclear whether these differences are attributed to the differences in the dimension descriptor, severity labels or a combination of both. Future qualitative work is recommended to establish the impact of the dimension descriptor on the reporting of problems.

Although the adolescents preferred the youth descriptive systems when considering the dimension descriptors and response options, the child friendly wording results in a “less severe descriptive system”. The youth version was similarly preferred in ET when considering preference regarding ease of use and best description of health state. However, ZA adolescents reported that both adult and youth descriptive systems were easy to use and described their health experience similarly. Consequently, the results are not interchangeable and not only affect the reported health state but by extension the preference of health state and the resultant value set. It is recommended that future research explore the acceptance of the youth descriptive system in adults and/or alternate options for aligning the descriptive systems.

Due to the limitations of the COVID-19 pandemic on recruitment at schools in ZA, there may be nonresponse bias. Although adolescents were explicitly instructed to complete the measures on their own without influence from others, there was no way to ensure this in the general and LSEN school samples in ZA. The use of different interviewers for school adolescents and those with health conditions was one of the ET study’s limitations. The data did not allow for assessment of responsiveness or change over time.

## Conclusion

Despite the overall high levels of agreement between EQ-5D instruments for youth and for adults, they do not provide identical results in terms of health state, from the same respondent. The differences were most notable for anxiety/depression and feeling worried/sad/unhappy. This was more notable with the transition between the Y-3L vs 3L and Y-3L vs 5L than the comparison between the Y-5L and 5L which may be due to more similar severity labels with translation into Amharic (ET). The cultural adaptation on the youth version in ET for having physical pain or discomfort (for example, itching, dizziness and feeling sick) reduces the severity of this dimension and future adaptations should consider this in terms of disparity between versions for transition. This cultural adaptation further limits the generalisability of the ET results to other Y-5L versions. It is recommended that future work investigate the differences in the Y-5L and 5L in English, the source language for translations. These differences in the way individuals respond to the various descriptive systems need to be taken into consideration for descriptive analysis, when transitioning between instruments, and when comparing preference-weighted scores.

**Table 1:** Sample characteristics

	Y-3L vs 3L	Y-3L vs 5L	Y-5L vs 5L
Country	South Africa		Ethiopia
Sample size			
Respondents	592		693
Sex, n (%)			
Female	325 (54.9)		352 (50.8)
Male	267 (45.1)		341 (49.2)
Age, n (%)			
13–15 years	228 (38.5)		223 (32.2)
16–17 years	269 (45.5)		353 (50.9)
18–19 years	95 (16.0)		117 (16.9)
Condition, n (%)			
Cardiac	36 (6.1)		91 (13.1)
Diabetes mellitus	107 (18.1)		75 (10.8)
Functional disability	27 (4.6)		55 (7.9)
General School	293 (49.5)		343 (49.5)
HIV	87 (14.7)		99 (14.3)
Respiratory disease	38 (6.4)		30 (4.3)
Other	4 (0.7)		

**Table 2:** Absolute difference in ceiling of each instrument for adolescents with a health condition

		EQ-5D-3L vs EQ-5D-Y-3L						EQ-5D-5L vs EQ-5D-Y-3L				EQ-5D-5L vs EQ-5D-Y-5L			
		**(ZA ** ***n*** ** = 298)**						**(ZA ** ***n*** ** = 298)**				**(ET ** ***n*** ** = 336)**			
		3L	Y-3L		3L vs Y-3L			5L	Y-3L	5L vs Y-3L		5L	Y-5L	5L vs Y-5L	
**Dimension**,** n (%)**		n (%)		n (%)		Δ n (%)	X^2^(p-value)	n (%)	n (%)	Δ n (%)	X^2^(p-value)	n (%)	n (%)	Δ n (%)	X^2^(p-value)
Mobility	1	250 (83.9)		251 (84.2)		−1 (−0.3)	0.01 (0.911)	254 (85.2)	251 (84.2)	3 (1.0)	0.18 (0.733)	244 (69.7)	243 (69.4)	1 (0.3)	0.01 (0.931)
Self-care/Looking after myself	1	275 (92.3)		278 (93.4)		−3 (−1.0)	0.23 (0.635)	276 (92.6)	278 (93.4)	−2 (−0.7)	0.10 (0.749)	285 (81.4)	280 (80.0)	5 (1.5)	0.28 (0.598)
Usual activities	1	226 (75.8)		227 (76.2)		−1 (−0.3)	0.01 (0.924)	220 (73.8)	227 (76.2)	−7 (−2.3)	0.44 (0.508)	246 (70.3)	242 (69.1)	4 (1.2)	0.12 (0.729)
Pain/discomfort	1	220 (73.8)		197 (66.1)		23 (7.7)	0.63 (0.427)	208 (68.8)	197 (66.1)	11 (3.7)	0.93 (0.334)	199 (56.9)	198 (56.6)	1 (0.3)	0.01 (0.937)
Anxiety/depression/ Feeling worried/sad/unhappy	1	211 (70.8)		180 (60.4)		31 (10.4)	**7.15 (0.007)**	208 (69.8)	180 (60.4)	28 (9.4)	**5.79 (0.016)**	198 (56.6)	199 (56.9)	−1 (−0.3)	0.01 (0.937)
Total ceiling	11111	134 (45.0)		111 (37.2)		23 (7.7)	3.67 (0.056)	135 (45.3)	111 (37.2)	24 (8.1)	**3.99 (0.046)**	148 (42.3)	152 (43.4)	−4 (−1.2)	0.10 (0.756)

Bold indicates significance with *p* < 0.05

**Table 3:** Discriminative power of each instrument by Shannon index (H′) and the evenness index (J′)

	EQ-5D-Y-3L vs EQ-5D-3L					EQ-5D-Y-3L vs EQ-5D-5L					EQ-5D-Y-5L vs EQ-5D-5L				
	**(ZA ** ***n*** ** = 592)**					**(ZA ** ***n*** ** = 592)**					**(ET ** ***n*** ** = 693)**				
	EQ-5D-3L		EQ-5D-Y-3L		Y-3L vs 3L	EQ-5D-5L		EQ-5D-Y-3L		Y-3L vs 5L	EQ-5D-5L		EQ-5D-Y-5L		Y-5L vs 5L
**Dimension**	H′	J′	H′	J′	ΔH′	H′	J′	H′	J′	Δ H′	H′	J′	H′	J′	ΔH′
Mobility	0.59	0.37	0.59	0.38	0.00	0.72	0.31	0.59	0.38	0.13	0.97	0.42	1.02	0.44	0.05
Self-care/Looking after myself	0.39	0.25	0.35	0.22	−0.04	0.46	0.20	0.35	0.22	0.11	0.64	0.28	0.71	0.31	0.07
Usual activities	0.87	0.55	0.85	0.54	−0.02	1.17	0.50	0.85	0.54	0.32	1.00	0.43	1.09	0.47	0.09
Pain/discomfort	0.97	0.61	1.08	0.68	0.11	1.24	0.54	1.08	0.68	0.16	1.29	0.56	1.34	0.58	0.05
Anxiety/depression/Feeling worried/sad/unhappy	1.27	0.80	1.31	0.83	0.04	1.69	0.73	1.31	0.83	0.38	1.40	0.60	1.47	0.64	0.07

Larger H′ value indicated higher discriminative power

**Table 4:** Known-groups validity for EQ-5D Level Sum score (LSS)

	EQ-5D-Y-3L vs EQ-5D-3L				EQ-5D-Y-3L vs EQ-5D-5L				EQ-5D-Y-5L vs EQ-5D-5L			
	ZA	3L	Y-3L	Y-3L vs 3L	ZA	5L	Y-3L	Y-3L vs 5L	ET	5L	Y-5L	Y-5L vs 5L
	*n*	Mean (SD)	Mean (SD)	Paired Difference (Mean ± SD)	*n*	Mean (SD)	Mean (SD)	Paired Difference (Mean ± SD)	*n*	Mean (SD)	Mean (SD)	Paired Difference (Mean ± SD)
**All Respondents**												
Total	592	6.3 (0.23)	6.4 (0.23)	0.1 (0.03)*	592	6.9 (0.36)	6.4 (0.23)	0.5 (0.06)*	693	6.8 (0.48)	7 (0.5)	0.2 (0.05)*
**Sex**,** n (%)**												
Female	325	6.3 (0.24)	6.4 (0.24)	0.1 (0.05)*	325	6.9 (0.39)	6.4 (0.24)	0.5 (0.08)*	352	6.9 (0.49)	7.1 (0.5)	0.2 (0.07)*
Male	267	6.3 (0.23)	6.4 (0.22)	0.1 (0.04)*	267	6.8 (0.32)	6.4 (0.22)	0.4 (0.09)*	341	6.8 (0.46)	6.9 (0.49)	0.1 (0.08)*
*F* statistics		0.13	0.02					0.02		0.09	0.40	
*p* value		0.720	0.894			0.438	0.894			0.77	0.528	
**Age**,** n (%)**												
13–15	228	6.3 (0.26)	6.5 (0.25)	0.2 (0.05)*	228	7 (0.41)	6.5 (0.25)	0.5 (0.08)*	223	7.3 (0.53)	7.4 (0.55)	0.1 (0.11)
16–17	269	6.2 (0.22)	6.4 (0.21)	0.2 (0.04)*	269	6.9 (0.32)	6.4 (0.21)	0.5 (0.08)*	353	6.5 (0.43)	6.8 (0.47)	0.3 (0.07)*
18–19	95	6.2 (0.23)	6.4 (0.22)	0.2 (0.1)	95	6.5 (0.31)	6.4 (0.22)	0.1 (0.14)	117	6.8 (0.45)	6.8 (0.43)	0.07 (0.12)
*F* statistics		0.24	0.23					0.23		4.21	2.26	
*p* value		0.788	0.797			0.186	0.797			**0.015**	0.106	
**Condition**,** n (%)**												
School	293	6.4 (0.22)	6.5 (0.22)	0.1 (0.04)*	293	7.2 (0.33)	6.5 (0.22)	0.7 (0.07)*	343	5.7 (0.25)	5.9 (0.27)	0.2 (0.06)*
Cardiac	36	6.1 (0.26)	6.3 (0.21)	0.2 (0.2)	36	6.6 (0.42)	6.3 (0.21)	0.3 (0.3)	91	7.9 (0.48)	8.4 (0.47)	0.5 (0.16)*
Diabetes mellitus	107	6.1 (0.24)	6.2 (0.23)	0.1 (0.08)	107	6.5 (0.34)	6.2 (0.23)	0.3 (0.1)	75	6.3 (0.27)	6.3 (0.33)	0.01 (0.12)
Functional disability	27	7 (0.33)	7.3 (0.32)	0.3 (0.2)	27	8.3 (0.57)	7.3 (0.32)	1 (0.5)	55	12.8 (0.37)	13.6 (0.36)	0.8 (0.27)*
HIV	87	5.9 (0.23)	6 (0.2)	0.1 (0.09)*	87	5.9 (0.26)	6 (0.2)	0.1 (0.13)*	99	5.7 (0.22)	5.8 (0.26)	0.1 (0.07)
Respiratory disease	38	6.5 (0.22)	6.9 (0.24)	0.4 (0.1)*	38	7.1 (0.33)	6.9 (0.24)	0.2 (0.2)	30	10.1 (0.55)	9.2 (0.6)	0.9 (0.6)
*F* statistics		2.95	4.78			6.06		4.78		91.56	91.59	
*p* value		**0.012**	**<0.001**			**<0.001**	**<0.001**			**<0.001**	**<0.001**	

*Wilcoxon signed-rank test, where a *p* < 0.05 was considered statistically significant, Bold indicates significance with *p* < 0.05

**Table 5:** Efficiency of the EQ-5D LSS and EQ VAS

Comparison			School Group	vs Cardiac disease	vs Diabetes mellitus	vs Functional disability	vs HIV	vs respiratory disease
EQ-5D-3L vs EQ-5D-Y-3L	South Africa	n	293	36	107	27	87	38
	EQ-5D-3L LSS	F	—	1.49	2.08	4.16	6.50	0.50
		P	—	0.222	0.150	**0.042**	**0.011**	0.482
	EQ-5D-Y-3L LSS	F	—	0.75	2.41	6.17	9.76	2.53
		P	—	0.388	0.121	**0.014**	**0.002**	0.113
	EQ-5D-3L EQ VAS	F	—	9.52	0.20	0.90	11.65	1.64
		P	—	0.002	0.658	0.342	0.001	0.201
	EQ-5D-Y-3L EQ VAS	F	—	8.50	0.12	1.77	10.17	2.61
		P		0.004	0.731	0.184	0.002	0.107
EQ-5D-5L vs EQ-5D-Y-3L	South Africa	n	293	36	107	27	87	38
	EQ-5D-5L LSS	F	—	1.88	6.01	4.81	20.99	0.08
		P	—	0.172	**0.015**	**0.029**	**< 0.001**	0.779
	EQ-5D-Y-3L LSS	F	—	0.75	2.41	6.17	9.76	2.53
		P	—	0.388	0.121	**0.014**	**0.002**	0.113
EQ-5D-5L vs EQ-5D-Y-5L	Ethiopia	n	343	91	75	55	99	30
	EQ-5D-5L LSS	F	—	75.74	11.14	497.06	0.03	123.79
		P	—	**< 0.001**	**0.001**	**< 0.001**	0.876	**< 0.001**
	EQ-5D-Y-5L LSS	F	—	87.77	4.62	514.23	0.23	65.23
		P	—	**< 0.001**	**0.032**	**< 0.001**	0.634	**< 0.001**
	EQ-5D-5L EQ VAS	F	—	13.30	0.29	77.30	0.14	6.26
		P	—	< 0.001	0.588	< 0.001	0.709	0.013
	EQ-5D-Y-5L EQ VAS	F	—	18.07	0.22	89.24	0.34	5.30
		P	—	< 0.001	0.639	< 0.001	0.561	0.022

Bold indicates significance *p* < 0.05. No VAS was collected for the EQ-5D-5L

**Table 6:** Preference for adult or youth instrument

	EQ-5D-3L vs EQ-5D-Y-3L				EQ-5D-5L vs EQ-5D-Y-5L			
	**(ZA ** ***n*** ** = 592)**				**(ET ** ***n*** ** = 693)**			
	Y-3L	3L	No Preference	Missing/ neither	Y-5L	5L	No Preference	Missing
**Easiest to use n (%)**								
Easy, understandable, straightforward, clear	87 (14.7)	31 (5.2)	143 (24.2)		139 (20.1)	62 (8.9)	31 (4.5)	
General preference, I don’t know or can’t explain		3 (0.5)			12 (1.7)	7 (1.0)	2 (0.3)	
Quicker/shorter	5 (0.8)	1 (0.2)	4 (0.7)		5 (0.7)			
More personal, relatable or relevant to me and my health	16 (2.7)	11 (1.9)	37 (6.3)		56 (8.1)	29 (4.2)	1 (0.1)	
Wording describes health well e.g., more detail, direct or specific	15 (2.5)	9 (1.5)	2 (0.3)		29 (4.2)	11 (1.6)	1 (0.1)	
Related to font – underlining, highlighting and/or bold	10 (1.7)	2 (0.3)						
Similar			73 (12.3)		1 (0.1)		51 (7.4)	
Better examples/options	2 (0.3)	2 (0.3)			3 (0.4)	5 (0.7)		
I am healthy, independent, and have no problems doing these things		1 (0.2)	7 (1.2)		10 (1.4)	5 (0.7)	1 (0.1)	
More interesting					4 (0.6)	1 (0.1)	1 (0.1)	
No reason	23 (3.9)	11 (1.9)	60 (10.1)		99 (14.3)	50 (7.2)	45 (6.5)	
Other	10 (1.7)	6 (1.0)	13 (2.2)		11 (1.6)	8 (1.2)		
Total	10 (28.4)	6 (13.0)	339 (57.3)	8 (1.4)	369 (53.2)	178 (25.7)	133 (19.2)	13 (1.9)
**Best describes my health experience**,** n (%)**								
Easy, understandable, straightforward, clear	23 (3.9)	11 (1.9)	24 (4.1)		50 (7.2)	26 (3.8)	4 (0.6)	
General preference, I don’t know or can’t explain	6 (1.0)	6 (1.0)	5 (0.8)		10 (1.4)	5 (0.7)	1 (0.1)	
Quicker/shorter					1 (0.1)			
More personal, relatable or relevant to me and my health	40 (6.8)	58 (9.8)	36 (6.1)		86 (12.4)	32 (4.6)	13 (1.9)	
Wording describes health well e.g., more detail, direct or specific	29 (4.9)	18 (3.0)	1 (0.2)		57 (8.2)	20 (2.9)	3 (0.4)	
Related to font – underlining, highlighting and/or bold		4 (0.7)						
Similar			106 (17.9)		1 (0.1)		50 (7.2)	
Better examples/options	5 (0.8)	6 (1.0)			3 (0.4)	3 (0.4)	1 (0.1)	
I am healthy, independent, and have no problems doing these things	7 (1.2)	4 (0.7)	1 (0.2)		15 (2.2)	3 (0.4)	2 (0.3)	
More interesting					2 (0.3)			
No reason	40 (6.8)	19 (3.2)	65 (11.0)		131 (18.9)	78 (11.3)	56 (8.1)	
Other	19 (3.2)	15 (2.5)	34 (5.7)		12 (1.7)	4 (0.6)		
**Total**	**269 (29)**	**141 (23.8)**	**272 (45.9)**	**10 (1.7)**	**368 (53.1)**	**171 (24.7)**	**130 (18.8)**	
**Preference considering dimensions descriptors**,** n (%)**								
Easy, understandable, straightforward, clear	96 (16.2)	48 (8.1)	68 (11.5)		55 (7.9)	30 (4.3)	3 (0.4)	
General preference, I don’t know or can’t explain	4 (0.7)	3 (0.5)	12 (2.0)		19 (2.7)	8 (1.2)	2 (0.3)	
Quicker/shorter	1 (0.2)	1 (0.2)			2 (0.3)	1 (0.1)		
More personal, relatable or relevant to me and my health	9 (1.5)	8 (1.4)	10 (1.7)		52 (7.5)	21 (3.0)	5 (0.7)	
Wording describes health well e.g., more detail, direct or specific	46 (7.8)	27 (4.6)	8 (1.4)		42 (6.1)	12 (1.7)	5 (0.7)	
Related to font – underlining, highlighting and/or bold	10 (1.7)	3 (0.5)						
Similar			32 (5.4)		9 (1.3)	3 (0.4)	31 (4.5)	
Better examples/options	5 (0.8)	8 (1.4)	3 (0.5)		4 (0.6)	2 (0.3)	1 (0.1)	
I am healthy, independent, and have no problems doing these things	3 (0.5)	1 (0.2)	2 (0.3)		6 (0.9)	3 (0.4)	3 (0.4)	
More professional/formal		4 (0.7)	1 (0.2)					
More interesting						4 (0.6)		
No reason	52 (8.7)	25 (4.2)	55 (9.3)		158 (22.8)	91 (13.1)	67 (9.7)	
Other	11 (1.9)	19 (3.2)	7 (1.2)		8 (1.2)	8 (1.2)	2 (0.3)	
Total	237 (40.0)	147 (24.8)	198 (33.4)	10 (1.7)	355 (51.2)	183 (26.4)	119 (17.2)	36 (5.2)
**Preference considering the response options**,** n (%)**								
Easy, understandable, straightforward, clear	74 (12.5)	32 (5.4)	50 (8.4)		73 (10.5)	27 (3.9)	3 (0.4)	
General preference, I don’t know or can’t explain	7 (1.2)	8 (1.4)	29 (4.9)		24 (3.5)	13 (1.9)	5 (0.7)	
Quicker/shorter	1 (0.2)	2 (0.3)	1 (0.2)		1 (0.1)	2 (0.3)		
More personal, relatable or relevant to me and my health	22 (3.7)	22 (3.7)	16 (2.7)		42 (6.1)	16 (2.3)	2 (0.3)	
Wording describes health well e.g., more detail, direct or specific	22 (3.7)	17 (2.9)	3 (0.5)		40 (5.8)	9 (1.3)	4 (0.6)	
Related to font – underlining, highlighting and/or bold	7 (1.2)	4 (0.7)	1 (0.2)					
Similar			47 (7.9)		2 (0.3)	2 (0.3)	36 (5.2)	
Better examples/options	5 (0.8)	7 (1.2)	1 (0.2)		6 (0.9)	1 (0.1)		
I am healthy, independent, and have no problems doing these things	4 (0.7)		1 (0.2)		7 (1.0)	3 (0.4)		
More professional/formal		4 (0.7)						
More interesting					7 (1.0)	2 (0.3)	1 (0.1)	
No reason	45 (7.6)	33 (5.6)	78 (13.2)		160 (23.1)	83 (12.0)	66 (9.5)	
Other	7 (1.2)	12 (2.0)	14 (2.4)		11 (1.6)	4 (0.6)	1 (0.1)	
Total	194 (32.8)	141 (23.8)	241 (40.7)	16 (2.7)	373 (53.8)	162 (23.4)	118 (17.0)	40 (5.8)

Preference between the Y-3L and 5L was not explored

### Electronic supplementary material

Below is the link to the electronic supplementary material.

Supplementary Material 1

## Data Availability

The datasets generated during and/or analysed during the current study are available from the corresponding author on reasonable request.
