# Supplementary material for: Transitioning between the EQ-5D youth and adult descriptive systems in a group of adolescents
Source: J Patient Rep Outcomes. 2024 Aug 12;8:93. doi: 10.1186/s41687-024-00770-4 (PMC11319571; doi:10.1186/s41687-024-00770-4)
Supplement: Supplementary file 1 — Supplementary Material 1 [file 41687_2024_770_MOESM1_ESM.docx]

Supplementary Table 1: EQ-5D Youth and Adult dimension descriptors and severity labels

| **Youth/ Adult dimension descriptors** | **English Severity labels** | | | | |
| --- | --- | --- | --- | --- | --- |
|  | Levels | Y-3L | **3L** | **Y-5L** | **5L** |
| *Mobility (walking about)*/  Mobility | 1 | no | no | no | no |
|  | 2 | some | some | a little bit | slight |
|  | 3 | a lot | confined to bed | some | moderate |
|  | 4 |  |  | a lot | severe |
|  | 5 |  |  | cannot | unable |
| *Looking after myself*/  Self-care | 1 | no | no | no | no |
|  | 2 | some | some | a little bit | slight |
|  | 3 | a lot | unable | some | moderate |
|  | 4 |  |  | a lot | severe |
|  | 5 |  |  | cannot | unable |
| *Doing Usual Activities (for example, going to school, hobbies, sports, playing, doing things with family or friends)*/  Usual Activities (e.g. work, study, housework, family or leisure activities) | 1 | no | no | no | no |
|  | 2 | some | some | a little bit | slight |
|  | 3 | a lot | unable | some | moderate |
|  | 4 |  |  | a lot | severe |
|  | 5 |  |  | cannot | unable |
| *Having Pain or Discomfort*/  Pain or Discomfort | 1 | no | no | no | no |
|  | 2 | some | moderate | a little bit | slight |
|  | 3 | a lot | extreme | some | moderate |
|  | 4 |  |  | a lot | severe |
|  | 5 |  |  | extreme | extreme |
| *Feeling worried, sad or unhappy* /  Anxiety/ Depression | 1 | not | not | not | not |
|  | 2 | a bit | moderately | a little bit | slightly |
|  | 3 | very | extremely | quite | moderately |
|  | 4 |  |  | really | severely |
|  | 5 |  |  | extremely | extremely |

Supplementary Table 2. Dimension agreement between instruments

|  | **South Africa (EQ-5D-3L vs EQ-5D-Y-3L)** | | | **Ethiopia (EQ-5D-5L vs EQ-5D-Y-5L)** | | |
| --- | --- | --- | --- | --- | --- | --- |
|  | **(n=592)** | | | **(n=693)** | | |
| **Dimension** | Cohen's kappa coefficients(95% CI) | Gwet's AC1 (95% CI) | Agreement (%) | Cohen's kappa coefficients(95% CI) | Gwet's AC1 (95% CI) | Agreement (%) |
| Mobility | 0.67(0.58,0.75) | 0.92(0.90, 0.95) | 92.9 | 0.72(0.66,0.77) | 0.90(0.88, 0.93) | 91.1 |
| Self-care/Looking after myself | 0.66(0.54,0.78) | 0.96(0.94, 0.97) | 95.9 | 0.68(0.60,0.76) | 0.93(0.91, 0.95) | 93.7 |
| Usual activities | 0.68(0.61,0.75) | 0.86(0.83, 0.89) | 88.5 | 0.65(0.59,0.71) | 0.87(0.84, 0.90) | 87.9 |
| Pain/discomfort | 0.76(0.71,0.81) | 0.86(0.83, 0.89) | 89.2 | 0.64(0.59,0.70) | 0.81(0.78, 0.84) | 83.3 |
| Anxiety/depression/Feeling worried/sad/unhappy | 0.59(0.53,0.65) | 0.68(0.63, 0.73) | 77.0 | 0.66(0.61,0.71) | 0.80(0.76, 0.83) | 82.4 |

*All results were significant at 0.01 level, CI confidence interval.*

*A Gwet’s AC of <0.2 was interpreted as poor agreement; 0.21–0.4 as fair; 0.41–0.6 as moderate; 0.61–0.8 as good and >0.8 as very good.*
